# Influences of Illumination Pretreatment on Soybean Oil Activated Clay Bleaching Effects and Soybean Oil Quality Evaluation

**DOI:** 10.3390/foods12051038

**Published:** 2023-03-01

**Authors:** Zhan Ye, Shufan Luo, Yaping Lv, Yuanfa Liu

**Affiliations:** 1 School of Food Science and Technology, Jiangnan University, No. 1800, Lihu Road, Wuxi 214122, China; 2 State Key Laboratory of Food Science and Technology, Jiangnan University, No. 1800, Lihu Road, Wuxi 214122, China; 3 National Engineering Research Center of Cereal Fermentation and Food Biomanufacturing, Jiangnan University, No. 1800, Lihu Road, Wuxi 214122, China; 4 National Engineering Research Center for Functional Food, Jiangnan University, No. 1800, Lihu Road, Wuxi 214122, China

**Keywords:** soybean oil, illumination pretreatment, edible oil bleaching, nutritional quality, fatty acid, lipid-soluble micronutrients

## Abstract

Visible light has been widely studied for possible applications in food industry as being a kind of clean energy. Presently, the influences of illumination pretreatment on soybean oil quality followed by conventional activated clay bleaching, including the oil color, fatty acid composition, oxidation stability, and micronutrient content, were investigated. Results demonstrated that the illumination pretreatment increased the color differences between the non-illuminated and illuminated soybean oils, which indicated that the light exposure could improve the decoloring effects. The fatty acids composition and the peroxide value (POV) and oxidation stability index (OSI) of the soybean oils showed little changes during this process. Although the illumination pretreatment affected the content of lipid-soluble micronutrients, including phytosterols and tocopherols, no significant differences could be observed (*p* > 0.05). Moreover, it showed that the illumination pretreatment showed significant effects for decreasing the following activated clay bleaching temperature, indicating the energy saving potential of this novel soybean oil decoloring process. The present study might provide new insights for developing eco-friendly and efficient vegetable oil bleaching technology.

## 1. Introduction

After being extracted from oil-bearing materials, crude oils should be refined prior to be used as edible oils. Edible oil refining involves multiple-step processes, which typically included degumming, alkali-refining, bleaching, and deodorization [1,2]. All of these processes were designed to remove both the endogenous and exogenous undesirable components or contaminants in crude oils, such as phospholipids, waxes, free fatty acids, pigments, off-flavors, and pro-oxidants, etc., so as to improve the quality of edible oil as well as storage safety [3,4].

The edible oil bleaching process is typically carried out after alkaline refining or degumming, and the primary focus of this process is to remove the different chromogenic substances and a wide variety of other impurities, most of which might impose adverse side effects on both the quality and stability of the final edible oil products [5,6]. An efficient edible oil bleaching process manages to remove certain pigments, such as carotenoids and chlorophylls, and decompose and partially remove oxidation products and contaminants, including soaps, trace metals, residual phospholipids, polycyclic aromatic hydrocarbons, etc. [7,8]. The edible oil bleaching process is typically conducted by using commercial industrial adsorbents, such as activated carbon, bleaching earth, silica hydrogel, etc., and the binding interactions between the chromogenic substances and the adsorbents are known as ‘adsorption’ [9,10]. Owing to its wide range of sources and excellent adsorption performance, bleaching clay has become the most used absorbent in edible oil refining industries [11]. Before being used, bleaching clay is usually activated via interaction with acids ranging from completely natural clays to highly acid-treated clays, which aims to improve the adsorbent activity and/or impurity removal, i.e., bleaching efficiency [12].

In the typical bleaching process, the amount of bleaching clay is about 2–5% of the oil, the bleaching temperature typically ranges from 90–125 °C, and the time for oil bleaching ranges from 15 to 45 min, with 20 to 30 min being most common. All these operation parameters largely depend on the oil quality (e.g., oil color, moisture content), characteristics of the adsorbents, and the processing technologies [1,10]. Operation pressure in the bleaching process also influences bleaching efficiency, and previous study has shown that bleaching effects are improved when absolute operating pressure in the bleacher is between 50 to 125 mmHg, which is beneficial for the removal of phospholipids, chlorophyll, carotenoids, and some other pigments [10,13]. Furthermore, the minimized interactions between the oil and air could result in lower peroxide values, anisidine values, and oil color, thus improving the overall oil quality [8,11]. Therefore, the traditional edible oil bleaching process is a typical high energy-consuming process. The adsorbent performance is predominantly regulated by the competitive absorption on the surface of the adsorbent due to thermodynamic limitations. The application of bleaching clay in this process might also bring many other shortcomings, including excessive neutral oil losses or oil filtration difficulties, which would decrease processing efficiencies. The spent bleaching clay also leads to disposal burden and might cause other environment problems.

In this regard, further studies are required to explore a sustainable, eco-friendly—and highly efficient—edible oil bleaching process. Fortunately, some preliminary research had been carried out [4,5,7,14]. One of the most widely explored techniques is the ultrasound-assisted bleaching process, which has been suggested to be able to improve oil bleaching efficiency; endogenous pigments can be removed to some extent even in the absence of absorbents due to heat and ultrasound pigment degradation effects. However, with this technique, the remaining or newly produced secondary oxidation products remain, which is considered the major disadvantage of this process [14]. Although the novel bleaching process that combined membrane filtration with adsorption was able to decrease secondary oxidation products in edible oils, lipid-soluble micronutrients, such as tocopherols, were also almost completely removed during this process, and the newly introduced pro-oxidation metal ions—including Cu, Pb ions—could also result in the lower oxidative stability of the final oil products [15].

Photocatalysis plays important roles in degrading organic colorants and pollutants; it was recognized as an eco-friendly and sustainable green catalysis method, and in recent years has gained much attention regarding its applications in different areas [16,17]. Although few studies have reported feasible applications of photocatalysis bleaching in the edible oil processing industries so far, from the limited data, it was suggested that light exposure could effectively reduce the chlorophyll content, with a degradation rate of 44.3% in a microalgae medium [18]. Chlorophylls are one of the major chromogenic substances in crude vegetable oils, and they should be removed due to their instable nature and the risk of them affecting the edible oil quality and stability [5]. The previous study showed that the absence of the Mg(II) ions in the central cavity of chlorophyll-α and chlorophyll-*b* did not result in changes in the electronic absorption spectra of chlorophyll, indicating that the chlorophylls in neutralized oil and the contained chlorophyll that lost Mg(II) ions in the central cavity still displayed similar absorption spectra and responses towards light exposure [19]. These implied the potential for photocatalysis application in the edible oil bleaching process.

Therefore, in the present study, the influences of visible light illumination pretreatment on the quality of soybean oil bleached by activated clay were studied, so as to explore the possibility of this clean energy being used as a supportive method in the edible oil bleaching process, thus improving oil quality. Firstly, the illumination pretreatment combined active clay bleaching process was designed, and the decoloring effects were also investigated by characterizing the CIELab color index and calculating the color difference of the different oil samples. Secondly, the influences of this new bleaching process on the fatty composition, as well as the oil oxidation stabilities, were studied. Finally, the oil-soluble micronutrients, including the different phytosterols and tocopherols, were analyzed by GC–MS and HPLC methods, respectively, so as to study their effects on the oil nutritional quality. The present study may provide both theoretical and practical foundations for the utilization of visible light illumination pretreatment for edible oil bleaching as a new, efficient, and eco-friendly method.

## 2. Materials and Methods

### 2.1. Materials

Neutralized soybean oil and bleaching clay were provided from Bohai Industrial Co., Ltd. (Binzhou, China). The reference standards, including the Supelco 37 Component FAME Mixture reference standard, tocopherols, and the 5-α-cholestane, 14% BF3 in methanol were all purchased from Sigma-Aldrich Chemical Co., Ltd. (St. Louis, MO, USA). The n-Hexane, isopropanol, and methanol were in HPLC grade, and were purchased from Sigma-Aldrich Chemical Co., Ltd. (St. Louis, MO, USA). All the other chemicals and reagents were of analytical or chromatographic grade and were purchased either from Sinopharm Chemical Reagent Co. Ltd. (Shanghai, China) or Thermo Fisher Scientific (Waltham, MA, USA). The Milli-Q water (Milli-Q Direct 8, Millipore, Burlington, MA, USA) was used in the present study.

### 2.2. Visible Light Exposure Pretreatment for the Soybean Oils

The visible light exposure pretreatment of the neutralized soybean oil samples was carried out in a multi-position photocatalytic reactor (Model: CEL-HXF300-T3), which was equipped with a Xenon light source system and was purchased from Beijing China Education Au-light Co., Ltd. (Beijing, China) (Figure S1). The condition parameters of the photocatalytic experiment process were maintained as follows: the visible light intensity in the range of 400–1000 nm was 352 mW/cm^2^; 450 g of neutralized soybean oil was divided into 9 groups and the illumination times were set as 0, 6, 12, 15, 18, 21, 22, 23, and 24 h. Subsequently, the pretreated oils were then bleached by activated clay.

### 2.3. Soybean Oil Activated Clay Bleaching

Batch adsorption experiments were carried out in a rotary evaporator at a constant speed, and every trial was performed according to the following steps: Prior to be used, the bleaching clay was firstly kept under 95 °C for 2 h for activation, then was cooled down to ambient temperature in an airtight desiccator. The neutralized soybean oil was pre-heated to 110 °C, and 3% wt (g/g, oil weight) of the activated clay was added. The process was conducted in a designed edible oil bleaching system (Figure S1), and the overall oil bleaching process was 40 min under different selected temperatures, with constant mechanical stirring at about 180 r/min (IKA@ RW 20, German). After the bleaching process, the oil–bleaching clay mixture was separated by vacuum suction filtration system equipped with a water-circulation multifunction vacuum pump (Ketai Co. Ltd., Zhengzhou, China). The bleached soybean oil was obtained from the top layer of the centrifugal tubes by centrifugation under 7500 r/min for 10 min (Sorvall ST 16R, Thermo Scientific, Waltham, MA, USA). The samples were used to for further quality analysis.

### 2.4. Bleaching Process Designed for Exploring the Possibiltiy of a Decrease in Processing Temperature

To explore the effects of the illumination pretreatment on the subsequent activated clay bleaching temperature, the low-temperature bleaching process was designated. Batch adsorption experiments were carried out as mentioned above, changing both of the illumination pretreatment conditions and the subsequent clay bleaching temperatures. Results from the soybean oil bleaching experiments with different illumination pretreatment conditions showed that 21 h of light exposure could significantly improve the decoloring effects without producing obvious side effects. Therefore, after 21 h of light exposure pretreatment, the soybean oil activated clay bleaching trials were performed under 110, 100, 90, 80, 70, 60, 50, and 40 °C. The content of the added bleaching clay was 3% wt (g/g, oil weight), and the bleaching process was kept for 40 min under the selected temperatures. The final oils were obtained from the top layer in the centrifuge tubes after being centrifuged under 7500 r/min for 10 min (Sorvall ST 16R, Thermo Scientific, Waltham, MA, USA). The bleached oil samples were used for further analysis.

### 2.5. Color Evaluation of the Bleached Soybean Oils

The color of different bleached soybean oils was evaluated by using a spectrophotometer (CS-820N, CHN Spec Co., Ltd., Hangzhou, China). The measurement of transmittance was carried out along with the visible spectrum (360–780 nm), geometry D/8 with a resolution of 0.01%. The color values were recorded as L*, a*, and b*, and the color differences between samples in pairs were evaluated as ΔE_00_, which were calculated according to the formula given by Equation (1), i.e., CIEDE2000 formula [20].

(1) $$∆E_{00}=\sqrt{{\left(\frac{∆L^{\prime }}{K_{L}S_{L}}\right)}^{2}+{\left(\frac{∆C^{\prime }}{K_{C}S_{C}}\right)}^{2}+{\left(\frac{∆H^{\prime }}{K_{H}S_{H}}\right)}^{2}+R_{T}\left(\frac{∆C^{\prime }}{K_{C}S_{C}}\right)\left(\frac{∆H^{\prime }}{K_{H}S_{H}}\right)}$$

In the Equation (1), the ${\Delta L}^{\prime }$, ${\Delta C}^{\prime }$, and ${\Delta H}^{\prime }$ were represented as lightness, chroma, and hue differences under CIELab color metric, respectively, all of which were calculated by the differences between the standard and sample in a pair. The $S_{L}$, $S_{C}$, and $S_{H}$ were the weighting functions and represented the lightness, chroma, and hue components, respectively. The $K_{L}$, $K_{C}$, and $K_{H}$ were the parametric factors that need to be adjusted according to the different viewing parameters, such as backgrounds, separations, etc., for the lightness, chroma, and hue components, respectively. $R_{T}$ was the interactive term between chroma and hue difference. All of the raw data read by the CS-820N spectrophotometer were analyzed and calculated with the online open-source platform.

### 2.6. Fatty Acid Composition Analysis by Gas Chromatography (GC)

The fatty acid compositions of the different soybean oil samples were analyzed by using a gas chromatography (GC) system (GC-2030 Nexis, Shimadzu, Kyoto, Japan), which was equipped with an auto-injector (Shimadzu, AOC-20i Plus), a Thermo Fisher Trace TR-FAME capillary gas chromatography column (60 m × 0.25 mm i.d. × 0.25 μm, Waltham, MA, USA) and a flame ionization detector (FID). The fatty acid methyl esters (FAMEs) were prepared according to the AOCS Official Method Ce 2-66 [21].

The multiple step GC analysis protocols were followed by our previous published work with some modifications [22]. In brief, the oven temperature was first held at 130 °C for 3 min, then was programmed to 200 °C by 5 °C/min, maintained for 10 min, and finally increased to 220 °C at the rate of 2 °C/min and maintained for 3 min. The temperature of the injector and the FID detector was held at 250 °C and 280 °C, respectively. The time of the total analysis procedure was about 40 min. The different fatty acids were identified by comparing their retention time with those in the FAME mixture reference standard, and the quantification of each fatty acid was calculated as the percent of each peak area relative to the totality of all peak areas.

### 2.7. Determination of the Soybean Oil Oxidation Stability Parameters

The peroxide value (POV) of the different soybean oil samples were characterized according to the AOCS official method Cd 8-53 [23], where the oil samples were treated in solution with acetic acid and chloroform solvent before being exposed to a potassium iodide solution, and the released iodine was titrated by the standard solution of sodium thiosulfate (0.01 M).

The Oxidative Stability Index (OSI) was determined by 892 Professional Rancimat (Metrohm, Herisau, Switzerland) according to the previous study, and the OSI was represented as the Rancimat induction time [24]. Briefly, 3.0 ± 0.1 g of the oil samples were tested under 120 °C, at an airflow rate of 20 L/h. According to the Metrohm’s recommendation, the temperature correction factor ΔT was set as 1.6 °C. All the operation processes followed the respective manufacturer instructions.

### 2.8. Sterols Composition and Tocopherols Content Analysis

#### 2.8.1. Phytosterol Quantification by Gas Chromatography–Mass Spectrometry (GC–MS)

The pretreatment of samples for phytosterol quantification was based on the method proposed by the previous researchers, with some modifications [25]. Briefly, 0.2–0.3 g of bleached soybean oil was dropped in a 20 mL glass test tube with a cap and was mixed with 0.5 mL of internal standard (5-α-cholestane, 0.1 mg/mL) and 3 mL of KOH-CH_3_CH_2_OH solution (2.0 M). Then, the mixture was subjected to water bath under 85 °C for 1 h. After being cooled down to room temperature, 2 mL of water and 5 mL of n-hexane were added, then the mixture was vortexed for 3 min. The supernatant was transferred to another eppendorf tube; while the residues were extracted by adding another 5 mL of n-hexane, the upper layer of solvent was taken out, and these two organic phases were merged into one glass tube. The solvent was dried by N_2_ blow, followed by the addition of 200 μL of BSTFA + TMCS (99: 1), and this mixture was held in the water bath at 75 °C for 30 min to facilitate the derivatization reaction. The BSTFA mixture was dried by N_2_ blow, and the extracts were re-dissolved in 200 μL hexane and filtered through a 0.22 μm membrane prior to the subsequent GC–MS analysis.

The GC–MS (Thermo Scientific, USA) was equipped with a DB-5 column (30 m × 0.25 mm i.d.,0.25 μm film thickness) and an FID. The GC programming conditions were as follows: the oven temperature was first held at 200 °C for 0.5 min, then increased to 300 °C by 10 °C/min, maintained for 18 min, and finally, the injector and detector were held at 280 °C. The injection volume was 1 μL. The splitter was opened at a 100:1 split ratio. The ion trap mass spectrometer was operated in the electron impact ionization (EI) positive mode, for which the instrumental parameters were set at the following values: filament emission current 80 A; transfer line, ion trap, and manifold temperatures were kept at 250 °C. The recording window was set between 50–550 m/z.

#### 2.8.2. Tocopherols Quantification by High-Performance Liquid Chromatography (HPLC)

The tocopherol content of the different soybean oil samples was quantified by HPLC (LC-20AT, Shimadzu, Kyoto, Japan), which was equipped with an ultraviolet detector (UVD) and a silica column (25 cm × 4.6 mm × 5 μm, Lichrospher SI). The analytical protocols were designed according to our previous study with some modifications [26]. Briefly, approximately 1.0 g of oil sample was dissolved in 10 mL of n-hexane and shaken vigorously, and was filtered by an 0.22 μm organic filter membrane. The diluted samples were injected into the column with a volume of 20 μL, and were eluted isocratically with mobile phase (n-hexane: isopropanol = 98.5: 1.5, *v*/*v*) with a flow rate of 1 mL/min at 30 °C.

Four kinds of tocopherols (i.e., campesterol, stigmasterol, β-sitosterol, and β-sitosterenol) were identified by the ultraviolet detector under 295 nm. The identification and quantification of tocopherol homologs were carried out by comparing the retention time and peak response of each sample with the corresponding analytical standards.

### 2.9. Statistical Analysis

All experiments were performed at least three times, and results were expressed as the mean ± standard deviation. Differences between experimental groups were compared by analysis of variance (ANOVA) (Tukey post-tests), with the significance level set at *p* < 0.05. All statistical analysis was performed using IBM SPSS Statistics, Version 20.0 (SPSS Inc., Chicago, IL, USA).

## 3. Results and Discussion

### 3.1. Influences of the Illumination Pretreatment on the Soybean Oil Activated Clay Decoloring Effects

**Figure 1:**
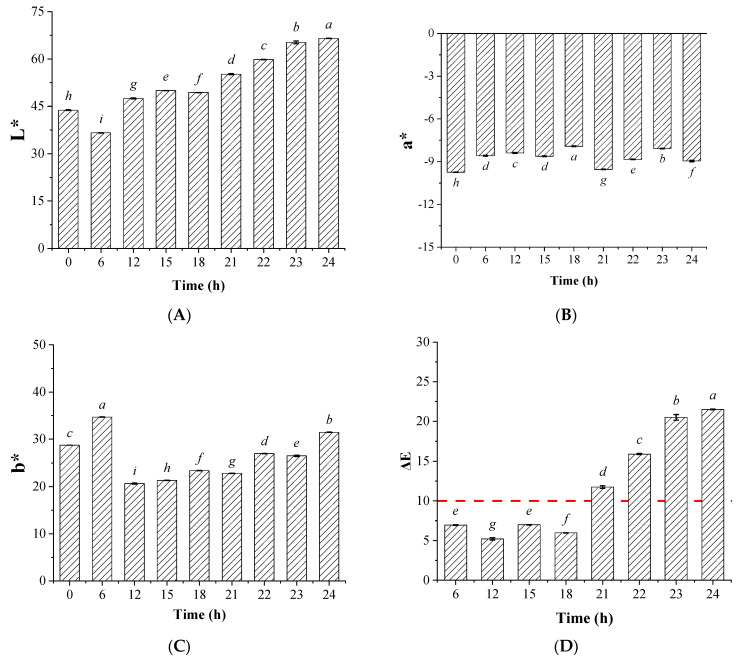
Color index (L*, a*, b*) (**A**–**C**) and the color-difference index (ΔE_00_) (**D**) of the activated clay bleached soybean oils with illumination pretreatment. The bars with different lowercase letters in the different groups indicate significant difference under *p* < 0.05; The group without illumination treatment (time for pretreatment was 0 h) was set as the control group.

The color or the transparency of the edible oils was one of the most important sensory attributes that showed significant influences on the consumers’ perception about the edible oil’s physical and chemical quality and was considered one of the most important quality evaluation parameters of commercial edible oil in China’s edible oil quality standards. The color index (L*, a*, b*) of the different soybean oils after the illumination pretreatment followed by activated clay bleaching process are displayed in the Figure 1.

The color index, which was calculated as L*, a*, and b* values, was widely accepted for the color evaluation of the liquid, solid, or semi-solid substances, such as edible oils. The L* value represented the lightness i.e., color black and white, and the a* value represented the colors red and green, while the b* value represented the colors yellow and blue. Furthermore, it was suggested that the a* and b* values indicated where the color fell along the red–green axis and yellow–blue axis. The higher a* value indicated the reduced level of green, while the higher b* value indicated an increased level of yellow [27]. As shown in Figure 1, compared with the control group, the L* value increased with the time of illumination pretreatment. After 24 h of the illumination pretreatment, the L* value of the bleached soybean oils reached 66.51 ± 0.04. As the higher L* value indicated a higher degree of lightness, thus, the increase in L* value over time might suggest that the illumination pretreatment showed potential for facilitating the activated clay bleaching process to improve the oil appearance. The a* value from the colorimeter represented the red–green value, in which the positive a* value indicated the redness, while the negative a* value represented the greenness. The a* value increased nonlinearly, which ranged from −9.74 to −7.91, whereas it could be concluded that the green levels were decreased for the oils that had undergone the illumination treatment. The b* value showed an initial decrease, then increase till 24 h, which implied that a longer time of light exposure might display adverse effects.

For further illustrating the color differences between the non-illuminated and illuminated groups, the color difference value was calculated according to the CIEDE2000 formula (Equation (1)), which was shown to better predict the suprathreshold color difference and indicate perception of color for the eyes [27]. The ΔE_00_ of the different groups are displayed in Figure 1D. It shows that the ΔE_00_ were all over 10 when the time of illumination pretreatment was over 21 h; moreover, a longer time of pretreatment also resulted in higher ΔE_00_ values. The previous study had suggested that an ΔE_00_ value over 10 indicated that there were significant differences of the oil colors between the different groups, and the higher ΔE_00_ value indicated more significant color difference [28]. From the present data, it can be seen that the illumination pretreatment led to the increase in lightness level and the decrease in green level. It could further be concluded that the light exposure also reduced the yellow or blue level of soybean oils after activated clay bleaching. Therefore, it indicated that visible light exposure improved the activated clay decoloring effects for the soybean oils, and moreover, these effects seemed to be more pronounced as the time of pretreatment prolonged.

### 3.2. The Changes in the Fatty Acids Compositions and Oil Oxidation Stability of the Soybean Oils

As indicated by the previous studies, a long duration of light exposure might affect the fatty acid composition of edible oils [29]. Presently, the fatty acid composition of the activated clay bleached soybean oils that were pretreated by the visible light exposure were analyzed (Table 1).

As shown in the Table 1, the five major fatty acids in the soybean oil in the different groups were ~13% palmitic (C16:0), ~5% stearic (C18:0), ~21% oleic (C18:1), ~50% linoleic (C18:2), and ~6% linolenic (C18:3ω3) acids, which could be counted for over 95% of the total. Moreover, no significant differences could be observed between the different groups (*p*> 0.05) although the time of illumination pretreatment prolonged. Specifically, the content of the three major unsaturated fatty acids, i.e., oleic acid (C18:1), linoleic acid (C18:2), and linolenic acids (C18:3), were 21.87%, 52.12%, and 6.35% in the untreated group, while those were 21.34%, 51.93%, and 6.50%, respectively, in the 24 h illumination treated groups, which indicated that the unsaturated fatty acids profiles also showed no significant changes although the soybean oil was pretreated by different light exposure conditions. The content of the total saturated fatty acids (SFA), mono-unsaturated fatty acids (MUFA), and the polyunsaturated fatty acids (PUFA) were calculated. Their contents showed no significant changes over the time of illumination pretreatment. There were few previous studies that had investigated the influences of the light exposure on decoloring effects of the edible oils, and the changes of fatty acid composition in this process were seldom discussed. However, as a kind of physical-assisted edible oil bleaching method, these results were similar with those reported in the previous study which showed that the microwave-assisted oil bleaching process showed no significant difference compared with the conventional bleaching process in terms of their fatty acid compositions [30]. These indicated that the physical-assisted edible oil bleaching methods showed good effects and might improve the overall oil quality.

**Figure 2:**
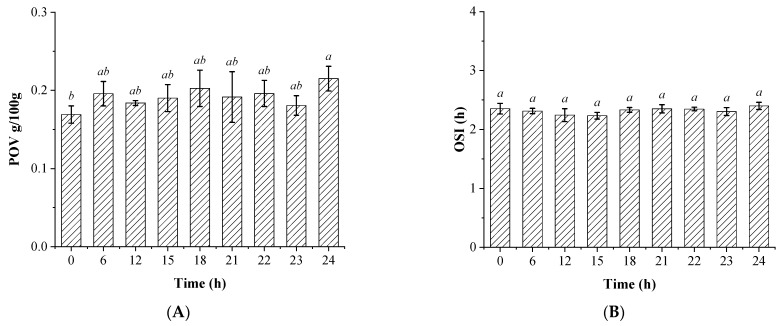
The peroxide value (POV) (**A**) and oxidative stability index (OSI) (**B**) of the soybean oils pretreated by visible light exposure prior to activated clay bleaching. The bars with different lowercase letters in the different groups indicate significant difference under *p* < 0.05.

However, for further investigating the quality of soybean oil after illumination pretreatment coupled with activated clay bleaching, the oxidation stability of the different oils was analyzed by characterizing the POV and oxidative induction time, and the results are shown in Figure 2. The results indicate that no significant differences could be observed regarding the POV of activated clay bleached soybean oils along with the time of illumination pretreatment from 0 to 23 h, although there was a significant increase for the oil with 24 h of illumination treatment. Furthermore, there were also no significant effects for illumination pretreatment on the OSI of the soybean oils within the time of observation (0–24 h). The previous study had suggested that the visible light exposure showed influences on the micronutrient content within the edible oils. In addition, the lipid-soluble bioactive micronutrients, such as polyphenols or tocopherols, also contributed to the oil oxidative stability due to their high antioxidant activity. Therefore, the edible oils with higher content of natural bioactive components typically displayed higher oxidative stability [29]. This was because the light exposure would significantly decrease the tocopherol, carotenoid, and chlorophyll contents compared with the oils kept in the dark. Further, it also significantly increased the production of secondary oxidation products as reported in the previous study [31]. However, these might also vary with the oil types; for example, there were differences between the extra virgin oil and commonly industrial bulk oils regarding the oxidation stability under the similar light exposure conditions. These might not only be attributed to the differences of contained oxidation precursor, but also partly due to the differences in fatty acid compositions of the oils.

For the present study, it could be concluded that the illumination pretreatment seemed to show no significant effects on the fatty acid composition and the oil oxidation stability of the soybean oils. This might be due to the fact that the light exposure led to the rapid degradation of the major chromogenic substances, such as chlorophylls, under the illumination conditions with visible-light (400–1000 nm) intensity at 352 mW/cm^2^, and therefore probably inhibited their mediated effects for promoting oil oxidation and triacylglycerols degradation [19,32,33].

### 3.3. The Changes in the Contents and Compositions of Sterols and Tocopherols

Since the light exposure was reported to affect the content of the micronutrients in the extra virgin olive oils in many previous studies, illumination pretreatment might also show influences on the compositions of the lipid-soluble micronutrients in the final soybean oils [29,31]. Therefore, the major micronutrients in the activated clay bleaching soybean oils that was pretreated with the different time of visual light exposure, including the phytosterols and the total tocopherols, were analyzed in detail.

As shown in the Table 2, although there were slight increases for the total sterol content in some soybean oil groups (e.g., the groups with illumination pretreated time of 6, 15, 18, 21, 22, and 24 h), there were no significant differences in both the total phytosterol content and the campesterol, stigmasterol, β-sitosterol, and β-sitosterenol sterol content between these groups (*p* > 0.05). As has been suggested in previous studies, these effects might be partly due to the photo-catalyzed hydrolysis of steryl esters, i.e., the newly produced sterols from the steryl esters hydrolysis compensate the sterol loss during the illumination treatment process [34,35,36].

**Figure 3:**
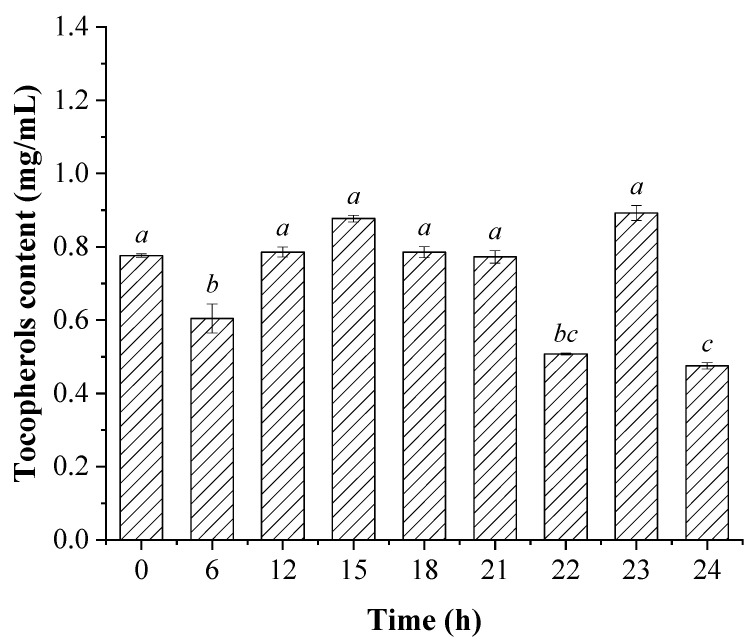
The tocopherols content in the soybean oils pretreated with visible light exposure prior to activated clay bleaching. Mean values with different letters over the bars indicate significant differences under *p* < 0.05 according to Tukey post-tests.

The total tocopherols content was characterized, which is displayed in Figure 3. As shown in the figure, the content of the total tocopherols was significantly decreased in the groups with 6, 22, and 24 h illumination pretreatment (*p* < 0.05), while the other groups showed no significant differences (*p* > 0.05). These results were also in accordance with the previous studies, which showed that tocopherols in the edible oils could be degraded under heat or light exposure conditions, and the degradation kinetics varied with the storage conditions or treatment methods [37,38,39]. It was suggested that the tocopherols content in the sunflower oils showed correlationships with the degree of oil oxidation, and the degradation of tocopherols increased with the increase in storage temperature. Moreover, the temperature showed more significant influences on the tocopherols’ degradation in the visible light exposure conditions than in dark conditions. However, the γ-tocopherol showed higher oxidation stability than the α-tocopherol within the oils in both the dark and light exposure conditions [40]. Another study also suggested that the α-tocopherol was markedly degraded with the increase in temperature between 180–260 °C and heating time between 20–80 min under reduced-pressure conditions (kPaA < 0 Pa) [41]. Therefore, the changes in the tocopherols content of the present study might be due to their photo-degradation and the structure transformation, however, further studies are still needed to elucidate the underlying mechanisms of the changes in both of their content and types in the light exposure process.

The phytosterols and tocopherols were the representative bioactive lipid-soluble micronutrients in the edible oils, both of which contributed to the nutritional value of the edible oils. They had been suggested to have many biofunctional roles for improving human health, such as serum lipid regulation, glycaemia improvement, cardiovascular diseases prevention, etc. [42,43]. Therefore, the present results indicate that the illumination pretreatment improved the soybean oil activated clay beaching effects, whereas, it did not significantly affect the content of these lipid-soluble micronutrients, which further implied that this novel edible oil decoloring process probably improved the edible oil refining effects without significantly affecting the nutritional value.

### 3.4. Effects of Illumination Pretreatment on the Soybean Oil Bleaching Temperature

**Figure 4:**
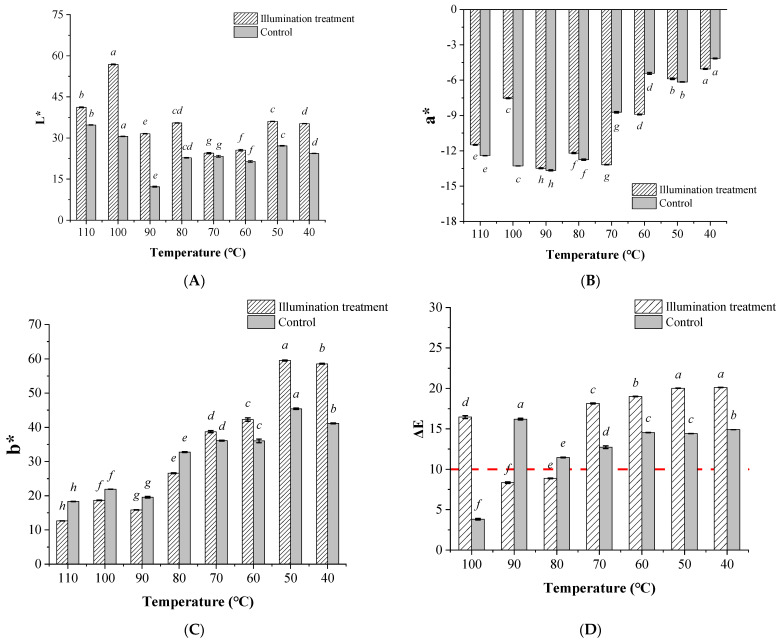
Color index (L*, a*, b*) (**A**–**C**) and the color-difference index (ΔE_00_) (**D**) of the bleached soybean oil (illumination pretreated groups and control group) under different bleaching temperatures. The data with different lowercase letters in the groups treated with different activated clay bleaching temperatures indicate significant difference under *p* < 0.05.

Typically, the edible oil bleaching process was conducted under 90–125 °C for 15 to 45 min with (typically for the continuous processing) or without (typically for the intermittent processing) vacuum conditions [10]. The high processing temperature was one of the most outstanding drawbacks of the conventional edible oil refining processing for producing nutritional edible oils, especially for the decoloring and deodorization processes [3,4]. The high processing temperature would also lead to the loss of many thermosensitive lipid soluble micronutrients, such as tocopherols, phytosterols, and polyphenols, etc. Therefore, to further explore whether the illumination pretreatment could decrease the temperature of the following activated clay bleaching process, the effects of the activated clay bleaching on the soybean oil color parameters with or without illumination pretreatment under different processing temperatures were compared. The color indexes of the different soybean oils were characterized (Figure 4).

This demonstrates that the L* values of the soybean oils were all significantly higher in the illumination pretreated groups than those in the untreated groups (control group) under certain bleaching temperatures (*p* < 0.05). It further indicates that the color of the soybean oil in the illumination pretreated groups seemed to become lighter. In the illumination pretreated groups, the highest L* value occurred for the soybean oil which was activated by clay bleached at 100 °C; moreover, the L* value generally showed a decreasing trend since the bleaching temperature decreased from 100 °C (Figure 4A). On the other hand, the a* value and b* value displayed uniform changes with the increase in the bleaching temperatures, and both were significant lower in the illumination treatment groups than those in the untreated groups (*p* < 0.05) (Figure 4B,C). Thus, it was suggested that the green and yellow color index were significantly changed after illumination pretreatment.

During the edible bleaching process, the temperature was about 100 °C, with 110 °C being the most typical—this might be considered as the ‘standard bleaching temperature’ [1,10]. Therefore, the color-difference index between the oils prepared under this standard bleaching temperature (110 °C) and those prepared under lower temperatures (i.e., 40–100 °C) after being treated or untreated with light exposure were compared (Figure 4D).

The results indicate that although the color-difference index (ΔE_00_) was over 10 (see the red dotted line) between the lower bleaching temperature groups (40–70 °C) and the 110 °C bleaching groups, the decoloring effects were not desirable, as indicated from the color parameters (Figure 4A–C, brighter, decreased in yellow intensity, and green intensity). From the results of the 100 °C activated clay bleaching groups, it showed that the decoloring effects of soybean oil were significantly improved, which further indicated that despite the bleaching temperature being decreased by 10 °C, the decoloring effects were not significantly affected. The results from the previous study suggested that the processing temperature was one of the most important parameters for edible oil bleaching [10], however, from the principle of the photocatalysis reaction, it was widely known that the excited-state chemistry varies from conventional ground-state pathways, and the photocatalysis was generally independent of temperature effects [44]. Therefore, the illumination pretreatment showed significant effects for decreasing the following activated clay bleaching temperature, without influencing the soybean oil bleaching efficiency and the oil quality. However, further studies about the mechanisms and kinetics of endogenous pigment degradation within soybean oils as well as changes in oil quality followed by the subsequent deodorization process are still needed.

## 4. Conclusions

In summary, in the present study, a new soybean oil bleaching process was proposed. The neutralized soybean oil was pretreated by Xenon light exposure with the wavelength of 400–1000 nm and the intensity of 352 mW/cm^2^, then was decolorized using conventional activated clay. The soybean oil decoloring effects as well as the oil quality were evaluated. The results demonstrated that the illumination pretreatment improved the soybean oil color indexes without significantly affecting the fatty acids composition and the oxidation stability, which was characterized by the POVs and OSIs. Moreover, the light exposure displayed influences on the lipid soluble micronutrients, including the phytosterols and the tocopherols, although no significant difference could be observed. Furthermore, the illumination pretreatment showed beneficial effects for decreasing the following activated clay bleaching temperature, without influencing the soybean oil bleaching efficiency and the oil quality, indicating the energy saving potential of this new decoloring assisting method. These findings might provide novel insights for the development of an efficient eco-friendly edible oil bleaching process. However, more work is still needed to uncover the underlying mechanisms and optimize the processing parameters in pilot scales.

## Figures and Tables

**Table 1:** The fatty acid composition of the activated clay bleached soybean oils.

Fatty Acid	Time of Illumination Treatment (h)								
	0	6	12	15	18	21	22	23	24
C16:0	12.62 ± 0.00 *^a^*	12.39 ± 0.00 *^a^*	14.58 ± 0.00 *^a^*	12.86 ± 0.00 *^a^*	12.47 ± 0.01 *^a^*	14.41 ± 0.03 *^a^*	12.48 ± 0.00 *^a^*	12.71 ± 0.00 *^a^*	12.55 ± 0.00 *^a^*
C16:1	0.42 ± 0.00 *^a^*	0.37 ± 0.00 *^a^*	0.36 ± 0.00 *^a^*	0.49 ± 0.00 *^a^*	0.40 ± 0.00 *^a^*	0.38 ± 0.00 *^a^*	0.37 ± 0.00 *^a^*	0.44 ± 0.00 *^a^*	0.53 ± 0.00 *^a^*
C17:0	0.42 ± 0.00 *^a^*	0.43 ± 0.00 *^a^*	0.40 ± 0.00 *^a^*	0.59 ± 0.00 *^a^*	0.42 ± 0.00 *^a^*	0.37 ± 0.00 *^a^*	0.46 ± 0.00 *^a^*	0.50 ± 0.00 *^a^*	0.68 ± 0.00 *^a^*
C17:1	0.28 ± 0.00 *^a^*	0.29 ± 0.00 *^a^*	0.30 ± 0.00 *^a^*	0.32 ± 0.00 *^a^*	0.41 ± 0.00 *^a^*	0.26 ± 0.00 *^a^*	0.36 ± 0.00 *^a^*	0.28 ± 0.00 *^a^*	0.43 ± 0.00 *^a^*
C18:0	4.61 ± 0.00 *^a^*	4.67 ± 0.00 *^a^*	4.79 ± 0.00 *^a^*	4.83 ± 0.00 *^a^*	4.75 ± 0.00 *^a^*	4.90 ± 0.00 *^a^*	4.67 ± 0.00 *^a^*	4.76 ± 0.00 *^a^*	4.71 ± 0.00 *^a^*
C18:1	21.87 ± 0.00 *^a^*	21.63 ± 0.00 *^a^*	20.91 ± 0.01 *^a^*	21.56 ± 0.00 *^a^*	21.62 ± 0.00 *^a^*	21.05 ± 0.01 *^a^*	21.74 ± 0.00 *^a^*	21.67 ± 0.00 *^a^*	21.34 ± 0.00 *^a^*
C18:2ω6	52.12 ± 0.00 *^a^*	52.76 ± 0.01 *^a^*	51.28 ± 0.01 *^a^*	51.76 ± 0.00 *^a^*	52.27 ± 0.01 *^a^*	51.06 ± 0.01 *^a^*	52.26 ± 0.01 *^a^*	51.83 ± 0.00 *^a^*	51.93 ± 0.01 *^a^*
C18:3ω6	5.90 ± 0.11 *^a^*	5.96 ± 0.17 *^a^*	5.54 ± 0.14 *^a^*	5.82 ± 0.07 *^a^*	5.81 ± 0.19 *^a^*	5.50 ± 0.35 *^a^*	5.87 ± 0.23 *^a^*	5.82 ± 0.14 *^a^*	5.97 ± 0.28 *^a^*
C18:3ω3	0.45 ± 0.01 *^a^*	0.52 ± 0.13 *^a^*	0.66 ± 0.07 *^a^*	0.50 ± 0.03 *^a^*	0.60 ± 0.26 *^a^*	0.77 ± 0.10 *^a^*	0.52 ± 0.12 *^a^*	0.51 ± 0.10 *^a^*	0.53 ± 0.10 *^a^*
C20:0	0.55 ± 0.00 *^a^*	0.26 ± 0.00 *^a^*	0.51 ± 0.00 *^a^*	0.55 ± 0.00 *^a^*	0.50 ± 0.00 *^a^*	0.60 ± 0.00 *^a^*	0.58 ± 0.00 *^a^*	0.78 ± 0.00 *^a^*	0.59 ± 0.00 *^a^*
C22:1	0.78 ± 0.00 *^a^*	0.72 ± 0.00 *^a^*	0.67 ± 0.00 *^a^*	0.73 ± 0.00 *^a^*	0.75 ± 0.00 *^a^*	0.71 ± 0.00 *^a^*	0.69 ± 0.00 *^a^*	0.71 ± 0.00 *^a^*	0.73 ± 0.00 *^a^*
∑SFA	18.20 ± 0.26 *^a^*	17.75 ± 0.97 *^a^*	20.29 ± 2.33 *^a^*	18.83 ± 0.42 *^a^*	18.15 ± 0.80 *^a^*	20.27 ± 2.69 *^a^*	18.19 ± 0.96 *^a^*	18.75 ± 0.24 *^a^*	18.54 ± 0.67 *^a^*
∑MUFA	23.34 ± 0.26 *^a^*	23.01 ± 0.13 *^a^*	2.24 ± 1.41 *^a^*	23.10 ± 0.18 *^a^*	23.18 ± 0.26 *^a^*	22.40 ± 1.25 *^a^*	23.16 ± 0.19 *^a^*	23.10 ± 0.26 *^a^*	23.03 ± 0.25 *^a^*
∑PUFA	58.47 ± 0.42 *^a^*	59.24 ± 1.01 *^a^*	57.48 ± 0.94 *^a^*	58.08 ± 0.36 *^a^*	58.68 ± 1.05 *^a^*	57.33 ± 1.44 *^a^*	58.65 ± 0.97 *^a^*	58.15 ± 0.39 *^a^*	58.43 ± 0.92 *^a^*

*^a^* The data with different lowercase letters in different groups indicated significant difference under *p* < 0.05.; The soybean oil was pretreated by visible light exposure prior to activated clay bleaching process.

**Table 2:** The phytosterols composition of the soybean oils pretreated by visible light exposure prior to activated clay bleaching.

Time (h)	Composition (mg/g)				
	Total Sterols	Campesterol	Stigmasterol	β-Sitosterol	β-Sitosterenol
0	3.61 ± 0.05 *^a^*	0.66 ± 0.01 *^a^*	0.78 ± 0.01 *^a^*	2.03 ± 0.07 *^a^*	0.14 ± 0.02 *^a^*
6	3.65 ± 0.55 *^a^*	0.65 ± 0.09 *^a^*	0.75 ± 0.06 *^a^*	2.09 ± 0.46 *^a^*	0.15 ± 0.02 *^a^*
12	3.40 ± 0.48 *^a^*	0.60 ± 0.05 *^a^*	0.88 ± 0.04 *^a^*	1.78 ± 0.13 *^a^*	0.14 ± 0.06 *^a^*
15	4.28 ± 0.15 *^a^*	0.85 ± 0.18 *^a^*	0.90 ± 0.07 *^a^*	2.38 ± 0.17 *^a^*	0.16 ± 0.03 *^a^*
18	4.23 ± 0.90 *^a^*	0.75 ± 0.18 *^a^*	0.95 ± 0.22 *^a^*	2.34 ± 0.47 *^a^*	0.19 ± 0.06 *^a^*
21	3.77 ± 0.09 *^a^*	0.69 ± 0.05 *^a^*	0.85 ± 0.01 *^a^*	2.08 ± 0.06 *^a^*	0.17 ± 0.03 *^a^*
22	3.73 ± 0.01 *^a^*	0.72 ± 0.01 *^a^*	0.79 ± 0.01 *^a^*	2.10 ± 0.01 *^a^*	0.12 ± 0.00 *^b^*
23	3.51 ± 0.18 *^a^*	0.67 ± 0.09 *^a^*	0.78 ± 0.05 *^a^*	1.90 ± 0.09 *^a^*	0.16 ± 0.03 *^a^*
24	3.84 ± 0.31 *^a^*	0.67 ± 0.08 *^a^*	0.81 ± 0.12 *^a^*	2.18 ± 0.14 *^a^*	0.18 ± 0.01 *^a^*

*^a^*^,^*^b^* The data with different lowercase letters in the different groups indicate significant difference under *p* < 0.05.

## Data Availability

The data presented in this study are available on request from the corresponding author. The data are not publicly available due to privacy restrictions.

## Supplementary Materials

The following supporting information can be downloaded at: https://www.mdpi.com/article/10.3390/foods12051038/s1, Figure S1: The schematic diagram of edible oil illumination pretreatment (A) coupled with activated clay bleaching (B) system (1-Xenon light generating system; 2-warm water bath, 3-heat transfer; 4-magnetic stirring generator; 5-temperature sensor; 6-soybean oil; 7-mineral oil bath; 8-heat generator; 9-mechanical stirring; 10-bleaching clay adding).

## Abbreviations

GC	gas chromatography
GC–MS	gas chromatography–mass spectrometry
HPLC	High Performance Liquid Chromatography
MUFA	monounsaturated fatty acids
OSI	oxidative stability indexes
POV	peroxide values
PUFA	polyunsaturated fatty acids
SFA	Saturated fatty acids
