# Supplementary material for: Influences of Illumination Pretreatment on Soybean Oil Activated Clay Bleaching Effects and Soybean Oil Quality Evaluation
Source: Foods. 2023 Mar 1;12(5):1038. doi: 10.3390/foods12051038 (PMC10001297; doi:10.3390/foods12051038)
Supplement: Supplementary file 1 [file foods-12-01038-s001.zip › foods-2154539-supplementary.pdf]

Supplementary materials

Figure S1

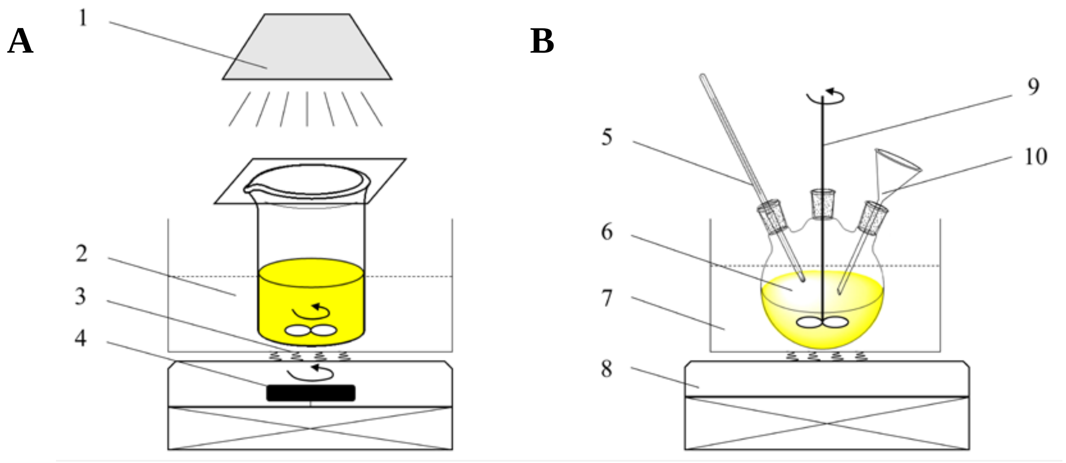

Figure S1. The schematic diagram of edible oil illumination pretreatment (A) coupled with active clay bleaching (B) system (1- Xenon light generating system; 2-warm water bath, 3-heat transfer; 4-magnetic stirring generator; 5-temperature sensor; 6-soybean oil; 7-mineral oil bath; 8-heat generator; 9-mechanical stirring; 10-bleaching clay adding).
